# Beyond task-space exploration: On the role of variance for motor control and learning

**DOI:** 10.3389/fpsyg.2022.935273

**Published:** 2022-07-19

**Authors:** Ernst-Joachim Hossner, Stephan Zahno

**Affiliations:** Institute of Sport Science, University of Bern, Bern, Switzerland

**Keywords:** motor variance, variability, sensorimotor learning, adaptation, learning mechanisms, meta-learning, dynamical-system theory, optimal feedback control

## Abstract

This conceptual analysis on the role of variance for motor control and learning should be taken as a call to: (a) overcome the classic motor-action controversy by identifying converging lines and mutual synergies in the explanation of motor behavior phenomena, and (b) design more empirical research on low-level operational aspects of motor behavior rather than on high-level theoretical terms. Throughout the paper, claim (a) is exemplified by deploying the well-accepted task-space landscape metaphor. This approach provides an illustration not only of a dynamical sensorimotor system but also of a structure of internal forward models, as they are used in more cognitively rooted frameworks such as the theory of optimal feedback control. Claim (b) is put into practice by, mainly theoretically, substantiating a number of predictions for the role of variance in motor control and learning that can be derived from a convergent perspective. From this standpoint, it becomes obvious that variance is neither generally “good” nor generally “bad” for sensorimotor learning. Rather, the predictions derived suggest that specific forms of variance cause specific changes on permanent performance. In this endeavor, Newell’s concept of task-space exploration is identified as a fundamental learning mechanism. Beyond, we highlight further predictions regarding the optimal use of variance for learning from a converging view. These predictions regard, on the one hand, additional learning mechanisms based on the task-space landscape metaphor—namely task-space formation, task-space differentiation and task-space (de-)composition—and, on the other hand, mechanisms of meta-learning that refer to handling noise as well as learning-to-learn and learning-to-adapt. Due to the character of a conceptual-analysis paper, we grant ourselves the right to be highly speculative on some issues. Thus, we would like readers to see our call mainly as an effort to stimulate both a meta-theoretical discussion on chances for convergence between classically separated lines of thought and, on an empirical level, future research on the role of variance in motor control and learning.

## Introduction: Some terminological hair-splitting

The present research topic is introduced with the description that, “human motor variability has been traditionally interpreted as an error of the system.” Although we do, in principle, perfectly agree with the statement, we would like to begin this conceptual analysis by pointing out that some room is left for further clarification. To this end, we propose to make a terminological distinction that helps to clarify this statement as well as our approach in this paper; and, generally, to be more precise when discussing issues of variability in the context of motor control and learning. Etymologically, the term *variability* can be traced back to the Latin words *variare* for *to change* and *habilitas* for *aptness* suggesting that “vari-ability” refers to an ability to change something, or to (intentionally) vary behavior to achieve particular goals. As we will see, in “traditional” accounts of motor control and learning, the ability to vary motor skills was a hot topic in motor-behavior research and regarded as a hallmark of skilled performance, hence, certainly not as an “error of the system.” However, what has indeed been neglected or classified as “errors” was (unintentional) *variance* at the level of movement execution and performance outcomes. Hence, we propose that a more precise description would be that: “human motor *variance*”—or even better, “the part of human motor variance that cannot be ascribed to human motor vari-*ability,*” “has been traditionally interpreted as an error of the system.”

However, even when accepting this terminological modification, we agree with the editors of the research-topic collection that there is still the need to consolidate the current understanding on the role of variance. So, after these introductory remarks (1), we will contribute to this consolidation by starting with a short overview on variance-related research in motor control and learning conducted over recent decades (2). This overview concludes in a deeper discussion on the function of variance for task-space exploration, which can be regarded as a fundamental learning mechanism independent of chosen theoretical perspective (3). However, as we will see, a more cognitive approach, in contrast to a dynamical-system framework, opens the door for the identification of further variance-based learning mechanisms. These mechanisms will be debated under the labels of task-space formation, task-space differentiation and task-space (de-)composition (4). Furthermore, the role of variance will be discussed in connection to mechanisms of meta-learning; specifically, with regards to the capabilities to handle noise, learning-to-learn and learning-to-adapt (5). The debate will be rounded up by a summarizing discussion, in which we argue to approach motor control and learning issues with a focus on specific learning effects caused by specific sorts of variance. This builds up to a call for investigating effects that can be observed on a behavioral level rather than restricting one’s perspective by theoretical concepts (6). Based on this discussion, we derive clear-cut empirical predictions for designing specific practice conditions to yield specific learning effects, especially in the practical context of motor learning in the domain of sports. These predictions are summarized in Table 1.

**Table 1:** The role of variance for different sensorimotor-learning mechanisms (in brackets: chapter number).

Task-space exploration (3)	With a local search in task-space exploration, noise affords the sensorimotor system slow and continuous learning without any further intervention
	In local search, a certain amount of noise is helpful to prevent the system from getting stuck in a local minimum of the task-space landscape.
	Adding noise might be required to escape a stable local minimum of the task-space landscape in order to induce “re-learning.”
	With a nonlocal search in task-space exploration, task variants should be practiced in a systematic manner while avoiding repetitions in a blocked schedule.
	Nonlocal task-space search can be induced by: discovery learning, adopting the constraints-led approach or providing learners with instructions that preferably relate to desired sensory consequences.
	Task-space exploration should be particularly promoted in regions of major as compared to minor importance for accomplishing the whole range of practically relevant task-goal variants.
	Task-space exploration should be guided into the direction of functional task-space regions that feature error tolerance and opportunities to exploit covariation or equifinality.
	In task-space exploration, only task-relevant variance should be considered while task-solution variants—specifically, variance in the task-irrelevant direction—should be particularly explored.
Task-space formation (4)	
	When task goals are missed in a fundamental manner, *de novo* learning should focus on task-space formation by providing multifarious support and reducing unwanted variance.
	Minimizing intended variance helps learners gain competence in regards to noise expectations and thus to identify basic movement structures.
Task-space differentiation (4)	
	Task-space differentiation, resulting from the identification of additional task-relevant control variables, would be best promoted by frequent switches between respective conditions.
	Frequent switches between task goals can be expected to decelerate the exploration of the corresponding task subspaces.
Task-space (de-)composition (4)	
	Accentuated variance in variables that form a functional task subspace should support learners in detecting functional (sub)structures that can be transferred to other tasks.
	Task-space (de-)composition can be further improved by practicing different tasks that include the same functional (sub)structure in order to let learners detect task-space distorting factors.
Handling noise (5)	
	The competence to estimate expected noise could be enhanced by exercises in which disturbing noise is added in order to make learners enforce their task goal against those perturbations.
	Adding more or less noise should foster the internal estimation of to what extent noise could either be actively controlled or be better handled by pursuing an impedance-control strategy.
Learning-to-learn/adapt (5)	
	The competence of learning-to-learn might be improved best by including not only a variety of differing tasks but also by inducing different ways of exploring respective task spaces.
	It seems plausible that the competence of learning-to-adapt is enhanced best by being frequently confronted with drastically changing task demands.

## A short history of variance-related research on motor control and learning

The previously debated description—that motor variance is regarded as a system error—derives from the early phases of motor-behavior research. During the so-called information-processing era, the human was basically conceptualized as a transmitter of information. Obviously, this conceptual starting point requires distinguishing between a “true” signal and signal-corrupting noise (Shannon and Weaver, 1949). In the stages-of-processing view (Marteniuk, 1976), noise should thus be expected to particularly impair information processing in the initial stage of sensation/perception and in the final stage of response execution. In this context, examining response execution in reciprocal finger tapping (Fitts, 1954), was able to empirically show that motor output variance can be described as a function of movement speed.

This overall negative view on noise also holds for the most prominent theory of the information-processing era, Schmidt’s (1975) schema theory. It should be noted, however, that at the same time, the advantage of variable over constant practice was repeatedly observed in empirical studies and underpinned by theoretical explanations. Indeed, a core prediction of Schmidt’s (1975) schema theory is that practicing variants of a generalized motor program improves schema formation by fostering interpolation between actually experienced program parameterizations. A related concept was suggested by the contextual-interference effect, an alternative explanation for the advantage of variable practice derived from the elaboration hypothesis. This explanation asserts that random practice of task variants, as opposed to blocked practice, leads to superior learning due to the opportunity to better compare these variants in short-term memory and thus to extract communalities and differences (Shea and Morgan, 1979). In contrast, the competing reconstruction hypothesis suggested that the random-practice advantage is attributed to the necessity to reconstruct the motor response repeatedly, which is thought to lead to a deeper processing of task-relevant information (Lee and Magill, 1983). In sum, this short overview shows that variability—as intended variance—was actually an intensely researched topic during the information-processing era of motor-control research; while input or output noise was generally seen as a performance-hindering feature of motor behavior.

In the 1980s, the information-processing approach was severely challenged by the observation that coordinated movement patterns reliably emerge as a function of organismic, environmental or task constraints (Newell, 1986). Most strikingly, this might even occur despite one’s intention; as was demonstrated by the inevitable change from a parallel to a symmetrical mode in coupled finger movements when the oscillation frequency exceeds a certain value (Kelso, 1981). This phenomenon has been formalized as a process of synergetic self-organization of the sensorimotor system, conceptualized in the metaphor of a potential landscape. For the finger movements, the landscape changes its form with increased frequency such that only the landscape’s symmetrical-mode attractor persists (Haken et al., 1985). As for the resulting phase transition from a parallel to a symmetrical mode, system-intrinsic variance is conceptually indispensable and empirically observable as an intensification of “critical” fluctuations around the transition point (Kelso et al., 1986). With this development, the notion of sensorimotor variance drastically gained importance in theoretical debates on sensorimotor coordination (Newell and Corcos, 1993). Interestingly, the resulting dynamical-system approach (for an overview, see Beek et al., 1995) – albeit not necessarily required on a conceptual level—has standardly been discussed as a companion to the ecological view on perception–action coupling (Gibson, 1979). This connection is apparently centered on a shared, more holistic understanding of human motor behavior. As this marriage implies, action concurrently results from and results in perception and vice versa. Consequently, both aspects are inextricably intertwined in a perception–action cycle (Kugler and Turvey, 1987). Therefore, sensorimotor learning would be best described as a process of searching the potential landscape, that is, the perceptual-motor workspace—in order to detect preferable regions as optimal solutions of the task at hand (Newell et al., 1989).

**Figure 1:**
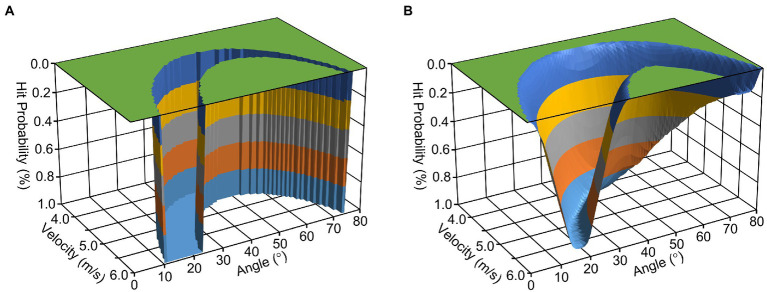
Task-space landscapes for dart throwing without **(A)** and with **(B)** considering sensorimotor noise.

The proposed approach of thinking in landscapes – or alternatively, spaces or manifolds – is illustrated in Figure 1A. Here, the task space for hitting a bull’s eye in dart throwing is depicted (as inspired by Müller and Loosch, 1999). For the sake of simplicity, the illustration is reduced to the dartboard’s vertical dimension, neglects aerodynamic aspects of the dart flight and is based on the assumption that the dart is constantly released in a distance of 2 m from the board, exactly at the height of the bull’s eye. Applying elementary physics, hits and no-hits are then determined just by the dart’s launch angle and velocity. As can be inferred from the task space’s form, the launch angle requiring the minimum velocity to hit the bull’s eye is 45^°^. However, as generating sufficient velocity is generally not a limiting factor in dart throwing (in contrast to, for instance, shot putting), the more interesting aspect of the illustration is the range of potential successful angle-velocity combinations. To the left of the valley in the region with smaller release angles, a wider range of velocity values can be combined to hit the bull’s eye; whereas to the right of the valley with larger release angles, a narrower range of velocity combinations are successful. Therefore, in this example, smaller angles are favorable over larger angles in terms of error tolerance (Müller and Sternad, 2004). From a dynamical-system perspective, this relationship constitutes a relevant constraint for the formation of an individual perceptual-motor workspace. However, other constraints need to be taken into account as well; for example, the organismic constraint that individuals differ in their capability to generate the high launch velocities required for extremely small launch angles.

Summing up, variance plays a remarkably different role in the historically opposed camps of information-processing and dynamical-system approaches to human motor behavior. Yet, it should be noted that since the peak of the so-called motor-action controversy more than 30 years ago (Meijer and Roth, 1988), dramatic conceptual re-orientations *within* cognitively rooted frameworks of motor control and learning have been observed. Most notably, there has been a stark shift from viewing motor control as a top-down determined product of linear information processing—as, for example, in motor-program theories—toward the idea of effect-related control. The latter perspective views motor control, in essence, as self-initiated transitions from current (perceived) states to desired (perceived) states. This shift toward effect-related control was mainly initiated by Prinz’s (1997) argument that it is impossible for a perceptual input to be directly translated into motor commands, as assumed in traditional information-processing models, due to the incommensurability of the respective codes. This fundamental insight gave rise to a revitalization of James’s (1890) ideo-motor principle that actions need to be planned in terms of desired—and thus perceivable—action effects.

Within the resulting ideo-motor conceptualizations of sensorimotor control, it is probably the internal-model approach (Wolpert and Ghahramani, 2000) that has yielded the most significant impact. In this theory, the ideo-motor notion of effect-relatedness prominently appears in the predictive function of an internal forward model (Jordan and Rumelhart, 1992). Specifically, the role of such forward models, which are developed and refined with experience, is to predict the sensory consequences of motor commands given a current (perceived) state. In the theory’s current variant of optimal feedback control (Todorov and Jordan, 2002), it is assumed that the sensorimotor system continuously refreshes its predictions for the transition from the current to the subsequent state. Thereby, and in sharp contrast to classical information-processing models, it is a core feature of optimal feedback control theory that not each and every state dimension need to be controlled (Todorov and Jordan, 2002: minimal intervention principle). Rather, intervening efferent commands are generated only if the internal forward model predicts that this intervention essentially contributes to the maintenance or the achievement of the currently desired state (for a closely related idea expressed in terms of affordances on the level of neural implementation, see Cisek, 2007). Notably, this implies that successful behavioral control crucially depends on reliable estimates of both current and desired states. To this end, the sensorimotor system is thought to optimally combine sources of information consistent with Bayesian principles (Körding and Wolpert, 2006). When determining the current state, noise estimations are used for weighting incoming (multi)sensory signals. In terms of one’s own motor commands, the noise in the execution is taken into account when selecting optimal task solutions (Trommershäuser et al., 2003). Consequently, a forward model for the dart-throwing task could be imagined as depicted in Figure 1B. Here, a task space as explained above has been overlaid by estimated noise in order to calculate probabilities (rather than mere physical dependencies) to achieve the desired goal of hitting the bull’s eye. This reorientation of the cognitively inspired branch of motor-control theory comes closer to the direction of dynamical-system approaches, rather than the traditional direction viewing motor control as a top-down determined product of central information processing.

## Variance for task-space exploration as a fundamental learning mechanism

By accepting the proposed convergence between the dynamical-system approach and recent versions of cognitive theories on motor control and learning, discussions on respective contributions of each to the explanation of motor behavior are no longer warranted. In turn, this creates room for the more interesting and applied question of how variance—whether it be inevitably or intentionally increased—could benefit motor learning. To this end, it can clearly be asserted that motor performance should improve with the more task-specific experiences gathered. This assertion would thus propose a progressive exploration of the corresponding task space (or, depending on the focus and the chosen terminology: the “perceptual-motor workspace”) as it was originally proposed by Newell et al. (1989) and has recently been theoretically and empirically updated by Pacheco et al. (2019).

In the present context, it is noteworthy that in the search strategy called “local search” by Newell et al. (1989), the role of noise is ambivalent. On the one hand, noise generally degrades performance; while on the other hand, it might by chance lead to the detection of a neighboring motor response that is even better suited to achieve the task goal. In this way, noise would result in a slow but continuous descent along the gradient of the task-space landscape and thus automatically provoke learning.

Furthermore, when descending along the gradient, a certain amount of noise is essential to prevent the system from getting stuck in a local minimum. This becomes particularly relevant if the minimum is sufficiently shallow to overcome its delimitating borders by such system-immanent noise. If this is not the case, adding more noise might be the most effective intervention. In sports, the issue of being stuck in a local minimum is well-known. For instance, when skiers find a stable solution to ride powder over a long phase of free practice, a technique with considerable layback may ultimately hinder maintaining control over the turns. If an instructor notices this error, he or she needs to take measures to initiate “re-learning”; for example, by making the skier feel the implications of riding powder while exaggeratedly laying either back or forward. Practicing these contrasting versions of a task solution can be conceptually understood as adding sufficient noise to overcome a local minimum. However, this does *not* imply that generally maximizing variations in practice as a matter of principle is beneficial—a recommendation known in the motor-learning literature as “differential-learning.” Since we are not able to find any comprehensible theoretical substantiation for “differential-learning methods,” we would prefer to skip a respective debate here (for details of this dispute, see Hossner et al., 2016a, and for a topic-related meta-analysis, Tassignon et al., 2021).

Beyond a local search, a nonlocal search strategy induces variations over a broader range of the task-space landscape (Newell et al., 1989). Besides stimulating “re-learning” as sketched above, this strategy can especially be applied in the initial phase of learning when the task can be solved in principle, but the initially chosen variants are far away from the task-space’s global minimum. In this case, when considering the empirical findings from contextual-interference research (which notably does not require the acceptance of cognitively-based explanations), it seems plausible that this search should be organized in such a way that repetitive, blocked practice of task variants are avoided (Lee and Magill, 1983: reconstruction hypothesis). Moreover, the variants should be presented in an order that facilitates the interpolation of the passed region of the landscape (Shea and Morgan, 1979: elaboration hypothesis). On this basis, we would recommend that learners be allowed to explore the task space in a more systematic than completely random manner (for empirical support for this recommendation, see Hossner et al., 2016a). Drawing on the ski-practice example above, this suggests organizing practice in a way that learners experience the consequences of variations in task-relevant dimensions (e.g., the distance of the skis, the degree of knee flexion, the extent of upward-downward movements etc.) in a systematic order, for instance, the backward–forward position on the skis from extremely laying back to extremely laying forward.

When conceptualizing motor learning as task-space exploration, instructors or teachers are challenged to apply promising methods to induce the generation of task variants in the learner’s behavior. To achieve this objective, three approaches are well-established in the world of sports. The first approach—termed discovery learning—is based on the assumption that learners are better able to detect their optimal way to solve a sensorimotor task than any external observer (e.g., Vereijken and Whiting, 1990). This means that learners should first and foremost be encouraged to explore the task space on their own. The second and slightly more prescriptive concept is known as the constraints-led approach (Davids et al., 2008). Here, the task space is (further) constrained in such a way that the learners are gently pushed into the direction of superior task solutions. Finally, and in an accentuated prescriptive manner, variants can be induced by explicit instructions. From a dynamical-system perspective, this third approach remains somewhat mysterious; since the challenging question arises as to how explicit information can be transformed into the language of task spaces in order to limit the search to a subspace. In this regard, the internal-model approach offers a straight-forward explanation: As instructions generate sensorimotor imagery together with the desired action consequences this imagery provides sufficient input for the movement to be formed (Hossner et al., 2020). Accepting this explanation would imply that instructions should preferably be provided in terms of desired sensory consequences, which are accessible to the learners themselves. Thus, all three learning approaches should help to induce nonlocal task-space exploration. In practical terms for the skiing example above, this would include motivating skiers to play with their position on the skis (discovery learning), letting them ride with rather open buckles to punish a non-centered position (constraints-led learning), or instructing them to feel more pressure at the shins than at the calves (perception-related instruction).

The more prescriptive a learning approach is the more the teacher needs to be sure of the superior regions of the task space. On the level of desired states, this demand can be achieved by ascertaining which regions play major or minor roles in accomplishing the whole range of practically relevant task variants. When practicing shooting in basketball, for instance, this means that the region for the free-throw distance to the basket should be explored more profoundly than other distances, which are experienced less frequently in ordinary games. When looking to motor execution, these differences between task-space regions with more or less fine-grained exploration result in the well-known especial-skill effect for “specificity embedded within generality” (Keetch et al., 2008).

If the relative importance of task-space regions is not that evident, the requirement to pre-determine superior regions in prescriptive learning approaches would be highly demanding from an observer’s perspective. This would be particularly challenging when the relevant space concerns the level of motor execution rather than directly goal-related performance. However, such a challenge could be facilitated by applying a thorough functional task analysis (Hossner et al., 2015). This concept can be illustrated in the context of the dart-throwing task illustrated in Figure 1: Reducing the overall coordination demands to two joints—namely the elbow and the wrist joint—can be expected to improve throwing precision, such that this variant is actually the only throwing technique utilized by darts experts. Hence, in the course of task-space explorations, learners should simply be instructed to avoid throwing variants that encompass, for example, shoulder movements or a posture in which the contralateral foot is positioned in front of the ipsilateral foot.

Beyond, the TNC-approach introduced by Müller and Sternad (2004) provides us with a powerful tool to decompose behavioral fluctuations and thus to determine superior regions of the task-space landscape. When applying this analysis to the darts-throwing task space illustrated in Figure 1B, it can be predicted that learners initially detect error-tolerant subspaces (T = tolerance). Specifically, the broad and deep left side of the depicted task-space valley is identified before noise is reduced as much as possible (N = noise). The first step in learning could thus be characterized as finding “stable solutions where intrinsic noise matters less” (Sternad et al., 2014). The second step then aims to exploit opportunities for covariation; which in our example would occur when deviations in the two determining variables of launch angle and velocity are compensated for one another (C = covariation). Most interestingly, the latter mechanism also encompasses the generation of “equifinal movement paths” (Müller and Loosch, 1999). Such a path in darts would be the (implicit) strategy to accelerate the dart with a corresponding change in movement angle, such that the achievement of the task goal becomes robust against the noise in the exact timing of dart launch.

The ideas of functional covariation and equifinality bring us to a final core concept regarding variance in sensorimotor learning in terms of task-space exploration: the uncontrolled-manifold concept (Scholz and Schöner, 1999). In this approach, the overall variance is decomposed into components that are either parallel or orthogonal to the task-solution subspace (i.e., in Figure 1B: to the bottom line of the valley). While the parallel component does not harm goal achievement and can thus be considered as “good variance,” the orthogonal component impacts achievement and is thus considered as “bad variance.” Interestingly, the same decomposition can be derived from the more cognitively inspired theory of optimal feedback control. From such a perspective, it has been shown that variance in the redundant, task-irrelevant direction is not only negligible but that pronounced reduction inevitably increases variance in the orthogonal, the task-relevant direction (Todorov and Jordan, 2002). These findings imply that, on the one hand, sensorimotor variance should only be considered if it (probably) affects performance error; while on the other hand, improving compensatory variability by an accentuated exploration of task-solution variants—as variance in the redundant, task-irrelevant direction—can be expected to particularly enhance the capability to optimally exploit existing variance. Nevertheless, it remains a matter of speculation whether further error amplification in the task-relevant direction might support the learning process; specifically, by clarifying the respective contributions of both dimensions to motor performance (for an overview, see Sternad, 2018).

## Further mechanisms: Variance for task-space formation, task-space differentiation, and task-space (de-)composition

As discussed before, task-space exploration can be regarded as a fundamental learning mechanism; be it from a dynamical-system perspective or, from the standpoint of optimal feedback control, in terms of exploring the landscape that constitutes an internal forward model. Further learning mechanisms can be derived from both theoretical frameworks, with these mechanisms similarly drawing on the landscape metaphor but exploiting it differently. To this end, in addition to task-space exploration, Hossner et al. (2020) distinguished task-space formation, differentiation and (de-)composition as important learning mechanisms. How variance affects learning based on these mechanisms will be discussed in the following paragraphs.

The first mechanism, task-space formation, can be mainly traced back to the applied issue of *de novo* learning of complex motor skills. The core problem regularly observed in such cases is that beginners fail to achieve the desired task goal not only by tendency (e.g., the dart has been successfully thrown but does not hit the board) but also in a fundamental way (e.g., a springboard diver fails to produce the forward rotation that is required for performing a desired somersault). Put into more theoretical terms: The executed movement does not yield an entry on the task-space landscape, and therefore task-space exploration is not feasible. Admittedly, as we will discuss later in this chapter, such cases of absolute *de novo* learning might be rare in real-world situations. However, they certainly occur; especially when control is demanded under completely novel conditions, may it be due to sports equipment (e.g., roller skates), the environmental medium (e.g., rough sea) or unnatural settings (e.g., running in virtual reality). Phenomenologically, these cases are often experienced as an almost entire loss of perception (e.g., in first sky-diving attempts), which can quite nicely be explained by optimal feedback control: The problem emanating from missing expectations is that they need to be generated by the forward model as a highly relevant contribution to current-state estimation. From the teacher’s or instructor’s side, *de novo* learning issues require the provision of basic support that can be put into practice through: (i) verbal hints like metaphors that call up sensory feelings related to task-goal achievement, (ii) appropriate schedules like repetitions of the very same task variant or (iii) the introduction of methodological steps like exercising only parts of the whole task. In all these cases, movement variance shows up as detrimental noise and further amplification does not seem to help the *de novo* learning. Notably, this statement can be extended from *de novo* learning to all situations in which learners must identify basic movement structures, because uncontrollable noise can apparently be better estimated if the variance due to intended task variations is minimized.

The second learning mechanism, task-space differentiation, has—to the best of our knowledge—gained only minor interest in the context of the task-space landscape metaphor to sensorimotor learning. This issue regards the fact that, in mainstream literature, the “task” to-be-solved is regularly introduced as a predetermined entity that seems to “exist” prior to being experienced by learners. Apparently, this assumption is problematic since tasks mandatorily need to be conceptualized as “tasks for someone.” Consequently, the focus should rather be placed on understanding how task spaces evolve with cumulated experience. To illustrate this point with an example from sports: Experienced skiers have definitely established different forward models for the consequences of motor commands when riding slalom skis vs. all-mountain wide skis. However, it does not make the slightest sense to assume that these internal models previously exist and “wait” for entries as soon as initial experiences with different types of skis are gathered. Quite the contrary, different variants of forward models need to be developed with specific practice from an initial common task space for skiing in general. Newell and Mayer-Kress (2001) perfectly acknowledge this issue and offer a “unified dynamical approach” as a solution. This suggests that behavioral changes should be considered on different time scales; in our context most notably, on the time scales of motor development, learning and adaptation. This approach was elaborated by Newell et al. (2003) using infant motor development as an example. It was demonstrated that prone progression can be described as a process of structuring a landscape on a rather long time scale. Attractor valleys for behaviors like lying down, chin up, creeping, sitting, standing and walking are developed over time and kept stable by forming a superimposed behavioral landscape. This conceptualization would ultimately come down to the idea that sensorimotor behavior is coordinated by one single overarching workspace—with (multidimensional) valleys for behaviors like creeping, skiing and throwing darts. Everyday experience, however, tells us that over the course of practice, one does not only improve in achieving certain task goals but also in switching between respective task (sub)spaces. Therefore, the process of, for instance, learning to differentiate between slalom or wide skis would probably not be described best as a progressively pronounced engraving of new valleys into the former general skiing attractor. This is due to the notion that deepened valleys would imply to make immediate switches between (sub)spaces more difficult. Such a notion certainly does not correspond to the everyday observation that switching from, for example, slalom to wide skis works increasingly reliably and instantaneously; that means, with a remarkable reduction of time needed for adaptation. From our view, this process should thus alternatively be conceptualized as the identification of additional task-relevant control variables that increase the task space’s dimensionality. These in turn allow for “shielding” the differentiated (sub)spaces against changes that, for instance, result from further experiences gathered in one (e.g., the slalom-ski subspace) or another (e.g., the wide-ski subspace) context. This process has thus been labeled as task-space differentiation by Hossner et al. (2020). Under the indication of “encoding of motor memories,” this process has received wide attention in force-field learning studies on cue-use in switching between different force fields (e.g., Howard et al., 2013). In regards to the question of inducing variance, it seems highly plausible that a frequent switch between relevant conditions should, on the one hand, promote the identification of additional task-relevant control variables but, on the other hand, can be expected to decelerate the exploration of the evolving task subspaces and vice versa. However, this prediction still considerably lacks of empirical support to date (for a promising approach in this respect, see Rohde et al., 2019).

The third learning mechanism proposed by Hossner et al. (2020), task-space (de-)composition, can be understood as a complementary process to the mechanism of task-space differentiation sketched above. The underlying issue centers on the enhanced capability to “shield” one task subspace against others, which would naturally diminish the chances of exploiting across-task transfer. However, this is an evident phenomenon in complex sensorimotor learning, for instance, when friends with different experiences in ball-throwing sports compete in darts for the very first time. As an initial approach to this contradiction—as it appears at first glance, Hossner et al. (2020) propose to assume that task subspaces are not only externally connected by differentiating variables but also internally structured on functional principles. To illustrate this idea again with the example of dart throwing: Given that the function relating launch angle and velocity to the outcome of a throwing movement (Figure 1) can be traced back to fundamental laws of physics, it would be worthwhile to utilize the knowledge gathered in experiencing this relationship in task A when confronted with a task B that requires throwing an object as well. Braun et al. (2009) refer to this phenomenon of across-task transfer as “structural learning”; wherein task-relevant structures are thought to be identified in the course of learning that, in turn, can be transferred to related tasks that overlap in exactly these features. In more implementational terms, the very same process can also be described as “modular decomposition” (Ghahramani and Wolpert, 1997). Evidently, such a decomposition process would be of significant help when one is confronted with a completely novel task, since task-space exploration can then start with a well-educated guess. This assumption, in turn, leads to the conceptual assumption that learning novel skills is regularly based on a transfer of functionally fitting subspaces from previous experience, and therefore real *de novo* learning is met in only rare cases. When it comes to structuring practice for facilitating the exploitation of across-task transfer, the objective would then be to support learners in identifying functional (sub)structures in their task spaces that can be potentially applied in the context of different tasks. Hossner et al. (2020) thus term the underlying learning mechanism task-space (de-)composition. Variance can be expected to support the process of identifying (sub)structures in the task space by pronouncedly exercising variants that refer to variables with functional relevance. In a practical context, this means that letting learners experience the consequences of either different launch angles or different launch velocities in isolation or in combination, while keeping all the other conditions constant, should allow them to detect the underlying structure. And in this way, the identified structure can be transferred to other tasks more easily. In addition, by introducing variations in different tasks that include the same structure, learning could be further enhanced by helping learners detect variables that distort the transferable structure in a predictable way; such as, for instance, variables related to the flight qualities of the object to-be-thrown.

## Variance for meta-learning: Handling noise, learning-to-learn and learning-to-adapt

Observant readers might have already noticed that the learning mechanism of task-space (de-)composition, discussed in the previous chapter, considerably exceeds the domain of mere skill learning because it prepares learners for successfully solving other tasks in the future by being able to transfer functionally relevant, clearly structured subspaces. Therefore, task-space (de-)composition can be regarded as kind of meta-learning. In this chapter, we will discuss three further mechanisms related to the domain of meta-learning and the role of variance in the acquisition of related skills; namely strategies to handle noise, to “learn-to-learn” and to “learn-to-adapt.”

Regarding the issue of handling noise, as discussed in previous chapters, variance in terms of unwanted (task-relevant) noise will naturally be reduced in the course of learning. This is due to: an improved assessment of perceptual variables, an enhanced estimation of the current state, a better image of the desired state in terms of expected sensory consequences and an increasingly fine-tuned specification of parameters as a result of a progressively refined exploration of the task space. However, inherent noise will never completely disappear. Therefore, as explained above, the deteriorating effects of remaining noise might be managed best by searching for error-tolerant regions of the task space (Sternad et al., 2014) and by continuously improving the competence to estimate noise-related effects on the movement outcome (e.g., Trommershäuser et al., 2003). The development of such competence would be best supported by minimizing the intended variance, as has been suggested in the context of the learning mechanism of task-space formation. However, it seems additionally plausible to expect further gains by conducting exercises in which disturbing noise is added in order to make learners enforce their task goal against those perturbations. Preferably, however, noise should only be added in dimensions where it also occurs in natural situations. Notably, practice for motor learning under such aggravating conditions is quite common in top-level sports. For instance, hockey coaches instruct their players to precisely take a shot on the goal while being roughly jostled by defending teammates. In addition, amplifying noise should also help learners develop the competence to distinguish between situations in which their skill level suffices to actively handle noise—by taking unwanted variance into account—and situations in which the noise is so provoking that it seems reasonable to switch to an impedance-control strategy (Hogan, 1985). In motor-control research, the latter strategy is commonly observed in force-field learning (Shadmehr and Mussa-Ivaldi, 1994). Applying such a strategy in this context, learners mainly increase the stiffness of co-contracting muscles to secure resistance prior to perturbations. This strategy is employed until the prediction of the perturbing forces can be progressively improved and ultimately, actively controlled *via* the production of appropriate counterforces (e.g., Franklin et al., 2003). Therefore, as a helpful tool, adding more or less noise during practice can foster the internal estimation of to what extent noise would be better handled actively or by pursuing an impedance-control strategy.

Classically, meta-learning is understood as the competence of learning-to-learn (Harlow, 1949). In relation to the variance-related sensorimotor-learning topic at hand, Pacheco et al. (2019) speculate on the relevance of not only the exploration of the task space *per se* but also the specific way in which it has been explored. In particular, they assume that the specific exploratory experience might serve as a sort of guideline when confronted with other tasks. This thereby encourages the learner to find and learn generalizable task solutions. From the internal-model perspective as illustrated in Figure 1B, we are happy to firmly second this suggestion. However, to the best of our knowledge, no research has been conducted so far on this issue of supposed meta-learning. In lieu of this, we may draw on anecdotal evidence from our own experiences as instructors in practical sports courses which repeatedly and reliably brought to our awareness that students with different individual learning histories approach novel tasks differently. In particular, as it seems to us, students highly experienced in sports games prefer to search for task solutions that allow them to exploit redundancies to free them from precise control of movement specifics. In contrast, experienced gymnasts tend to rather constrain their individual workspace in the direction of a single stable solution, which becomes apparent in more precise but also more rigid movements than those observed in game-experienced students. However, this anecdotal evidence is of course highly speculative and should be taken as a mere plausibilization of the assumption by Pacheco et al. (2019). Nonetheless, if this assumption would prove reliable, variance for motor learning in sports education should not only encompass different sports (which would develop a breadth of transferable functional structures) but also include different ways of exploring the respective task-space landscapes in order to improve the competence of learning-to-learn.

Closely related to, though distinctly separate from, the learning-to-learn issue is the hypothetical competence of learning-to-adapt that would refer to non-permanent changes in performance on a shorter time scale. Such a competence was addressed by Hossner et al. (2016b) under the label of “capability to adapt” in the course of a debate on the potential advantages of the induction of maximum fluctuations in motor learning. In this context, it was argued that a maximization of inter-trial noise over acquisition might exert detrimental effects on the learning rate, though improve the capability to quickly adapt to changing conditions. To emphasize, this competence should not be confused with the introduced mechanism of task-space differentiation, which takes on the assumption that adaptation is accelerated due to the identification of task-relevant variables resulting from previous experience. In contrast, what is meant here is the competence to acquire (implicit) meta-strategies of how to quickly adapt to unknown conditions or task goals without knowledge of predictable specifics of the observed perturbations. It seems plausible that this competence would be enhanced best by being frequently confronted with drastically changing task demands. Although this is once again highly speculative, we still find this idea worthwhile to be pursued further.

## Conclusion: A call for specificity in research on variance for motor control and learning

The conclusions that can be drawn from this paper are twofold. First, when evaluating the summary of hypothetical effects of variance on sensorimotor learning in Table 1, it becomes obvious that variance is neither generally “good” nor generally “bad” for sensorimotor learning. Rather, specific forms of variance can be expected to cause specific effects in regards to specific learning mechanisms. For instance, as it is speculated in Table 1, an increase of variance in form of unwanted noise would support: (i) escaping a local minimum in the task-space landscape to induce “re-learning,” (ii) enhancing the skill to enforce an intended task goal against perturbations and (iii) fostering meta-learning in terms of an improved capability of learning-to-adapt. At the same time, however, accentuated noise supposedly hinders learners from gaining competence in assessing noise expectations and thus identifying basic movement structures. Consequently, rather than investigating whether variance is either “good” or “bad” for sensorimotor learning, we would like to argue that future research should focus more on specific cause-effect relationships. Particularly, specific forms of variance should be evaluated with respect to their specific effect on specific learning mechanisms. If pursuing such a research strategy results in the finding that, for example, a specific form of variance enhances learning mechanism A but impairs learning mechanism B, this finding should then not be disparaged as contradictory evidence. Rather such outcomes should be appreciated as useful differential information for designing optimal practice conditions for sensorimotor learning.

Notably, this first conclusion further extends to an appeal to design empirical research on variance for motor control and learning more so on the basis of low-level operational than high-level theoretical terms. If, for instance, variable vs. constant conditions are investigated in basketball shots, it would not make any sense to expect more or less of an advantage from frequently changing the distances to the basket if the very same intervention has been derived from either classic information-processing theories (e.g., motor-schema formation; Schmidt, 1975) or the dynamical-system approach (e.g., searching perceptual-motor workspaces; Newell et al., 1989). In contrast, theoretical terms – particularly the distinction of specific learning mechanisms, should rather come into play on a higher level. This means theoretical terms should be utilized to derive predictions of which specific learning intervention should result in which specific effects on permanent performance changes which implies that—on the lower level of practical concepts—one and the same intervention could yield positive effects for competence A but negative effects for competence B.

This last-mentioned point brings us to our second conclusion; namely that—at least in our view—the time is ripe to overcome the outdated controversy between motor and action approaches in motor-behavior research that flared up more than 30 years ago (Meijer and Roth, 1988). As already highlighted above, it should be noted that proponents of the “motor camp” have considerably shifted over recent decades, acknowledging that not all details of observable movements can be “prescribed” in a top-down manner and that movements are necessarily controlled in terms of intended and anticipated action effects. When taking the—more cognitively inspired—theory of optimal feedback control (Todorov and Jordan, 2002) as an example, these re-orientations are implemented by an internal forward model (plus an internal pseudo-closed loop) such that behavioral control is ultimately conceptualized as moving from the currently (estimated) perceived state to a future (estimated) perceived state defined by the desired task goal. Obviously, this notion of control as a transition from perception to perception—that is more or less affected by one’s own motor commands—brings current motor theories much closer to a Gibsonian view on perception–action coupling (Gibson, 1979) than it was the case for classic information-processing theories of motor control and learning (e.g., Schmidt, 1975). In Figure 1, this convergence of historically incompatible theoretical frameworks is demonstrated; the landscape metaphor not only holds for an appropriate task description from a dynamical-system perspective but also suits to illustrate the structure of an internal forward model. As a matter of course, one still might vehemently debate whether the qualification of forward models as being located “internally” or whether and to what extent these models “represent” the world. For the practice of sensorimotor learning, however, we expect that in most cases these issues—that are rooted in disparate philosophical stances—result in differences regarding only the preferred terminology and/or level of analysis rather than in actually competing hypotheses for designing practice for motor learning. Therefore, we call for proponents of the “action camp” to make a similar shift as their more cognitively orientated colleagues. A good starting point for mastering this challenge might be to consider the empirically substantiated proof that bimanual coordination is evidently controlled in the space of perceptual consequences rather than in the space of relative joint angles (Mechsner et al., 2001). This, in turn, would imply that coordination is achieved in terms of anticipated—specifically, forward-modeled—action effects.

Ultimately, the resulting search for converging lines and mutual synergies in the explanation of motor behavior phenomena comes down to an acceptance of the inheritance left by Bernstein (1967). On the one hand, the work of Bernstein (1967) certainly focused on autonomously made contributions of lower levels of a sensorimotor control hierarchy but, on the other hand, also underlined the necessity to acquire a “model of the future” on a higher control level. This takes on the idea of how the current state of the system can be best transferred into a desired state, or in other words, how a reliable internal forward model is developed. We thus curiously look forward to becoming aware of common endeavors to bridge obsolete theoretical gaps—with respect to the role of motor variance for motor control and learning or beyond, which will inevitably enrich future research on sensorimotor behavior.

## Author contributions

All authors listed have made a substantial, direct, and intellectual contribution to the work and approved it for publication.

## Funding

Open access funding was provided by the University of Bern.

## Conflict of interest

The authors declare that the research was conducted in the absence of any commercial or financial relationships that could be construed as a potential conflict of interest.

## Publisher’s note

All claims expressed in this article are solely those of the authors and do not necessarily represent those of their affiliated organizations, or those of the publisher, the editors and the reviewers. Any product that may be evaluated in this article, or claim that may be made by its manufacturer, is not guaranteed or endorsed by the publisher.
