# Erratum: Beyond task-space exploration: On the role of variance for motor control and learning

**DOI:** 10.3389/fpsyg.2023.1180626

**Published:** 2023-03-15

**Authors:** Frontiers Production Office

**Affiliations:** Frontiers Media SA, Lausanne, Switzerland

**Keywords:** motor variance, variability, sensorimotor learning, adaptation, learning mechanisms, meta-learning, dynamical-system theory, optimal feedback control

An omission to the funding section of the original article was made in error. The following sentence has been added: “Open access funding was provided by the University of Bern.”

The original article has been updated.

